# Ultra-High Through-Cure of (Meth)Acrylate Copolymers via Photofrontal Polymerization

**DOI:** 10.3390/polym12061291

**Published:** 2020-06-04

**Authors:** Catharina Ebner, Julia Mitterer, Paul Eigruber, Sebastian Stieger, Gisbert Riess, Wolfgang Kern

**Affiliations:** 1 Department of Polymer Engineering and Science, Chair in Chemistry of Polymeric Materials, Montanuniversitaet Leoben, 8700 Leoben, Austria; catharina.ebner@unileoben.ac.at (C.E.); julia.mitterer@unileoben.ac.at (J.M.); paul.eigruber@unileoben.ac.at (P.E.); wolfgang.kern@unileoben.ac.at (W.K.); 2 Department of Polymer Engineering and Science, Chair of Injection Moulding of Polymers, Montanuniversitaet Leoben, 8700 Leoben, Austria; sebastian.stieger@unileoben.ac.at

**Keywords:** photofrontal polymerization, photopolymerization, ultra-high through-cure, photobleaching, (meth)acrylate, macromonomers, TPO-L, type I photoinitiator

## Abstract

Photopolymerization offers substantial advantages in terms of time, temperature, energy consumption, and spatial control of the initiation. The application however is strongly limited due to the constrained penetration of light into thick films. Strategies to overcome the problem of limited curing depth, as well as to improve the curing of shadow areas, involve dual curing, frontal polymerization, and upconversion of particles. Whereas excellent results have been accomplished applying photofrontal polymerization on a theoretical level, few studies report on practical applications achieving high curing depth within short time. This study aims to investigate the potential of photofrontal polymerization, performed only with photoinitiator and light, for the fast and easy production of several-centimeter-thick (meth)acrylic layers. Monomer/ initiator systems were evaluated with respect to their optical density as well as photobleaching behavior. Moreover, depth-dependent polymerization was studied in specimens of varying monomer ratio and photoinitiator concentration. When an ideal photoinitiator concentration was selected, curing up to 52 mm in depth was accomplished within minutes.

## 1. Introduction

**Figure 1:**
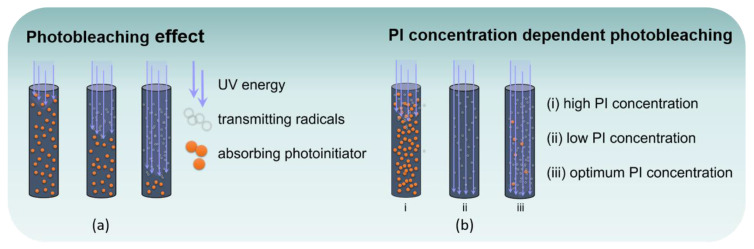
(**a**) Schematic representation of photofrontal polymerization, i.e., photobleaching during light exposure: absorbing photoinitiator (PI) molecules decompose into transparent products leading to an increase penetration depth. (**b**) PI concentration dependent penetration depth and curing: (i) high PI concentration resulting in increased absorption prevents deep penetration, (ii) low PI concentration enables high penetration depth with less radicals generated in the bottom region thus leading to low through-cure, (iii) with PI concentration in the optimum range, photobleaching allows light to penetrate into the bottom region still with enough radicals generated to achieve homogenous curing.

Ultraviolet and, more recently, visible light have been used in industry for about 60 years to cure polymeric materials such as films, printing inks, coatings, dental composite resins, photoresists, as well as 3D printed parts in additive manufacturing [1,2,3,4,5,6]. In a light-induced radical polymerization, an initiator molecule is excited to higher energy levels by absorption of light, resulting in alpha or beta cleavage of a labile bond or abstraction of hydrogen, thus generating free radicals promoting polymerization in regions accessible to light. The fact that only areas exposed to light are cured allows controlled spatial polymerization and production of complex micro-scale structures with smooth surface [7,8]. Nevertheless, low penetration depths, caused by decreasing intensity according to Beer‒Lambert law, greatly limit the application potential. Depending on the absorptivity of the formulation, the photoinitiator (PI) type, and the actinic source, layers with typically up to 50 µm or at the very most a few millimeters thickness are commonly polymerized [9]. Various strategies have been developed to circumvent this limitation. Thermal frontal polymerization, for example, applies complementary initiators activated by heat generated in exothermic reactions. Thus, light induced polymerization reaches shadow areas and is able to cure large batches [10,11,12,13,14]. Upconverting (nano) particles, on the other hand, act as “internal lamps” by emitting photons within the bulk material. Liu et al. [15,16,17] employed this mechanism to cure a 8.4 cm thick thiol-ene system. Another promising and convenient method to cure systems consisting only of monomer(s) and photoinitiator involves photobleaching. Also known as photofrontal polymerization, this effect is based on the property of photoinitiators whose decay products exhibit an absorption behavior differing greatly from that of the initiator itself [18,19]. As a consequence, light penetrates deeper into a formulation in the course of irradiation and higher depth of cure (DOC) values are achieved (Figure 1a). Photoinitiators with photobleaching capability include substituted titanocenes, benzophenone, acylphosphine oxides (e.g., the trimethylbenzoyl phosphine oxide (TPO) family), and also 1,2-diones such as camphorquinone and its derivatives, which have gained interest due to their low volatility and good absorption in the visible spectrum [5,20,21,22,23]. According to previous studies, high curing depths of 10‒13 cm may be achieved with photobleaching systems (PBS) [9,24]. Despite these promising approaches, only few studies did attempt to optimize photobleaching systems.

**Figure 2:**
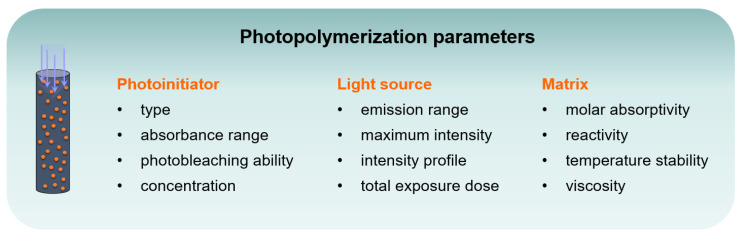
Key parameters for optimum curing (depth and photobleaching) in a light-induced radical polymerization.

An efficient implementation, however, would offer significant benefits: Systems that achieve high DOC values due to photobleaching do not necessarily require additional initiators, expensive additives, or rare earths. Moreover, casting molds may ensure an efficient manufacturing of components in large quantities. In addition, high intensity curing systems are already available, which operate more sustainably, e.g., microwave power. State-of-the-art simulations provide an excellent basis for optimization [25,26,27,28,29,30]. Miller et al. presented a mathematical description of photopolymerization systems with photobleaching initiators [26]. According to their simulation, the spatial propagation of the initiation front is increased by enhancing the quantum yield and reducing the molar absorptivity of the monomers or the photolysis products. In investigating the dependency of curing depth on PI concentration, Lee et al. pointed to the presence of an optimum PI concentration for a given irradiation dose [31]. Two opposing effects play a role in this context: with a higher PI concentration the rate of radical formation will increase, but, in parallel, absorption of the formulation is raised, thus hindering penetration. Therefore, an optimum PI concentration should be low enough to allow deep penetration upon photobleaching but still high enough to generate a sufficient amount of initiating radicals for uniform curing (Figure 1b). Additional factors to be considered when designing photobleaching systems include the processing and optical characteristics of the matrix (i.e., molar absorptivity) as well as the emission output and intensity of the light source, which need to be reconciled with PI absorbance and concentration (Figure 2).

Further, optimization of the intensity profile may be decisive for successful curing: Hayki et al. demonstrate that for full photobleaching a sigmoidal intensity profile leads to homogeneous radical formation from top to bottom, whereas for partial photobleaching an exponential intensity profile is beneficial [32]. Considering the mentioned parameters, the present study aims to explore the potential of photobleaching systems for the fast and easy production of thick (meth)acrylic layers in bulk. With a low optical density strategy in combination with TPO-L as highly efficient photobleaching initiator, a rapid homogenous through-cure of up to 52 mm thick specimens is accomplished. Offering resource-saving production and easy processing, polytetrahydrofuran methacrylates are particularly well suited for this purpose. Moreover, they are thermally stable and therefore tolerant to high intensity irradiation. Photopolymerizable systems consisting of long chain polytetrahydrofuran dimethacrylate as the crosslinker, 2-ethylhexyl acrylate as the reactive diluent, and TPO-L as the photoinitiator are characterized in this study.

## 2. Materials and Methods

### 2.1. Materials

Polytetrahydrofuran with an average molecular mass of 2900 g/mol (PTHF_2900_), 2-ethylhexyl acrylate (2-EHA), methacrylic anhydride (MAA), as well as 2,6-di-tert-butyl-cresol (BHT) were purchased from Sigma Aldrich (Vienna, Austria) and used without further purification. 2,4,6-Trimethylbenzoylphenyl phosphinate (Omnirad TPO-L) was provided by IGM Resins (Walwijk, The Netherlands).

### 2.2. Polyol Modification

**Figure 3:**
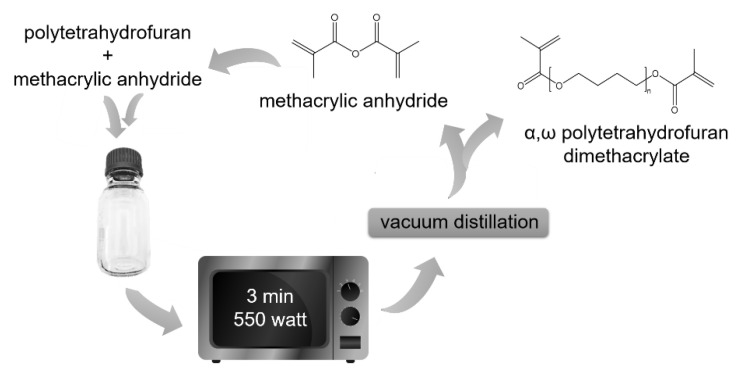
Microwave-induced transesterification of PTHF_2900_ with methacrylic anhydride followed by vacuum distillation, separating the highly temperature stable PTHF_2900_-DM from recyclable methacrylic anhydride and methacrylic acid.

PTHF_2900_ modification was performed in bulk via microwave-assisted transesterification (3 min at 550 W for 150 g polyol) [33] with two fold molar excess of methacrylic anhydride. Subsequent vacuum distillation separate α,ω-polytetrahydrofuran-dimethacrylate (PTHF_2900_-DM) from unreacted methacrylic anhydride and methacrylic acid formed during the reaction. A total of 500 ppm of BHT, was added to the mixture to prevent unwanted polymerization. Modification and purity were monitored by ^1^H-NMR using a Varian 400 (Varian, Waldbronn, Germany) and ATR FT-IR (Vertex 70, Bruker, Ettlingen, Germany) spectroscopy. The developed procedure is completely solvent free and enables fast and easy recycling of excess methacrylic anhydride and methacrylic acid (Figure 3).

### 2.3. Preparation of Photobleaching Systems (PBS)

**Figure 4:**
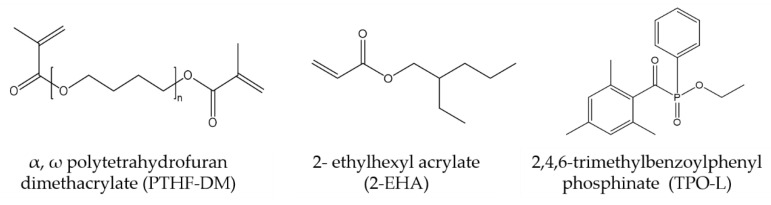
Components of photobleaching systems: difunctional PTHF_2900_-DM and 2-ethylhexyl acrylate in combination with TPO-L.

Formulations containing PTHF_2900_-DM, 2-EHA, and TPO-L were subjected to ultrasonication (water bath, 15 min, 50 °C) and mixed to homogeneity using VM-200 (StateMix, Winnipeg, Canada) prior to curing and characterization (Figure 4).

### 2.4. UV-Vis Spectroscopy and Photobleaching Experiments

UV-Vis spectra of monomers and PBS were recorded using a Cary 50 UV-Vis spectrophotometer (Varian, Waldbronn, Germany). Determination of extinction (*E*) followed Beer‒Lambert law:

(1) $$E=log_{10}\left(\frac{T_{0}}{T}\right)$$

where *T*_0_ denotes the incident transmittance and *T* is the measured transmittance. The molar absorptivity (*ε*) is determined according to:

(2) $$\varepsilon \;\left(L\;mol^{-1}cm^{-1}\right)=\frac{E}{c\times d}$$

where *c* is the molar concentration and *d* is the path length. To monitor photobleaching, PTHF_2900_-DM containing 0.15 wt % TPO-L was repeatedly subjected to a defined exposure dose using a Light Hammer 6 curing system (Fusion UV Systems, Gaithersburg, Maryland, US) equipped with semi-elliptical reflector, LC6B benchtop conveyor and a medium pressure mercury lamp (emission range between 250 and 470 nm). The applied intensities and exposure dose in the sample plane were determined using UV Power Puck^®^ II radiometer (EIT Instrument Markets, Sterling, Virginia, US) (Table 1). The total duration of the exposure was no more than a few minutes. A standard precision cuvette of 44 mm height (Hellma, 100-QA, one centimeter path length) with a removable light-shielding cover (several layers of aluminum foil) served as the mold. After each exposure cycle, transmittance was recorded in the spectral range between 200 and 800 nm. Self-made cuvette inserts enabled height-resolved collection of spectra (i.e., at 2, 22, 32, and 42 mm) to follow the progressing depth-dependent photobleaching from top to bottom. The light-shielding effect of the cover was tested beforehand by covering the top side with foil as well and applying the specified exposure. An unpolymerized (liquid) PBS proved that lateral exposure is negligible. Calculations of depth-dependent relative PI decay were based on changes in transmittance (*T*) at 375 nm according to:

(3) $$Relative\;PI-decay=\frac{\Delta T}{\Delta T_{max}}\times 100=\frac{T-T_{0}}{T_{max}-T}\times 100$$

with *T*_0_ as the absorbance before exposure and *T*_max_ the maximum absorbance obtained after full exposure.

### 2.5. Photopolymerization and Characterization of Curing Behavior

Photobleaching systems for specimen production received a pretreatment as described in detail in Section 2.3. Polymerization was accomplished in cylindrically shaped Teflon^®^ molds of 52 mm depth and one centimeter inner diameter with a total exposure of 20.8 J cm^−2^ after 9 runs (Table 2). Again, a cover of several layers aluminum foil prevented lateral exposure.

**Figure 5:**
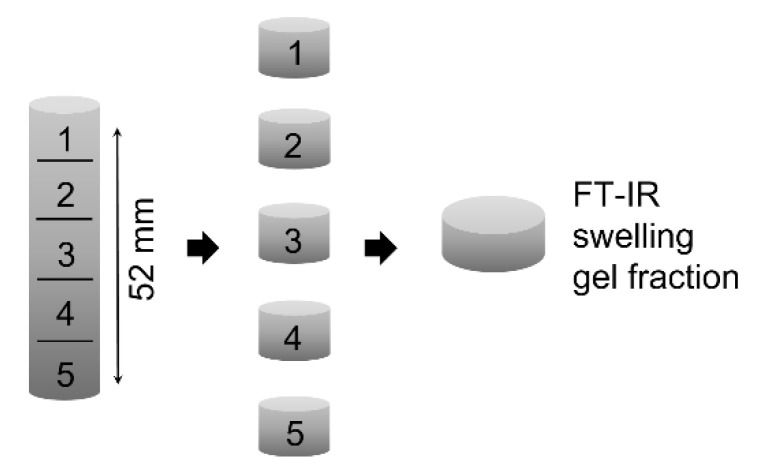
Sectioning of test specimen to evaluate depth-dependent curing.

To assess the uniformity of polymerization, the specimens were sliced into five equal pieces and analyzed separately by swelling tests (including the determination of gel content) as well as FT-IR spectroscopy (Figure 5).

FT-IR spectroscopy was performed with an ATR (attenuated total reflection) accessory on a Vertex 70 (Bruker, Ettlingen, Germany) spectrometer in transmission mode with accumulation of 16 scans. In order to detect the mass loss of hydrophilic and hydrophobic components, ethanol and toluene served as solvents for swelling experiments. Specimen parts were allowed to swell for 240 h at 40 °C. The mass related solvent-uptake (swelling ratio *Q*) was determined according to:

(4) $$Q=\frac{w_{s}-w_{i}}{w_{i}}$$

with *w*_i_ as initial mass of the polymeric network and *w*_s_ the mass of the swollen network. From the mass remaining after vacuum drying at 110 °C the gel-content was determined according to:

(5) $$Gel-content\;\left(\%\right)=\frac{w_{d}}{w_{i}}\times 100$$

with *w_d_* as mass of the remaining dry network.

## 3. Results and Discussion

### 3.1. Optical Characteristics of Monomers and Photobleaching Systems

**Figure 6:**
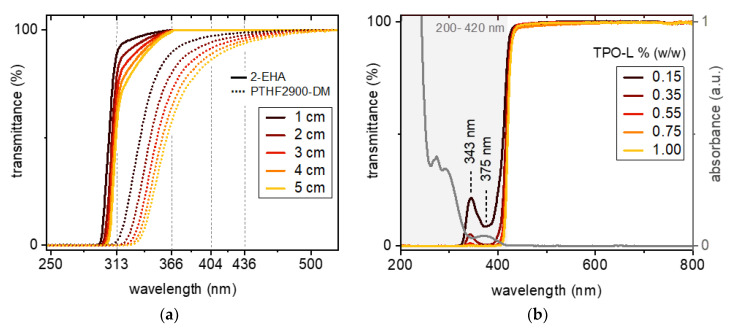
(**a**) Pure monomers, PTHF_2900_-DM and 2-EHA, transmit light down to wavelengths of 330 and 305 nm, respectively, at one centimeter sample depth. The attenuation of light with increasing layer thickness is more pronounced with PTHF_2900_-DM. (**b**) Upon TPO-L addition, transmittance decreases, starting from 420 nm. Formulations with low TPO-L concentration (up to 0.55 wt %) display maxima (345 nm) and minima (375 nm) in transmittance, reflecting the characteristic absorbance of TPO-L.

The absorptivity of monomers represents a major factor for deep curing and affects the ability of formulations to photobleach. Any absorption by the monomer, especially in the long or medium wave UV (UVA, UVB) or Vis region, would hamper deep penetration as it competes with the absorbance of the photoinitiator. Pure monomer formulations of PTHF_2900_-DM and 2-EHA are highly transmissive for light up to a wavelength of around 330 and 305 nm at one centimeter layer thickness (Figure 6a). Considering the emission spectrum of a conventional medium-pressure mercury lamp, which was used in this study, the monomers allow the strong UVA and Vis emission lines at 366 (*i*-line), 404 (*h*-line), and 436 nm (*g*-line) to penetrate almost unhindered. Being also transparent to the relatively strong UVB output line at 313 nm, 2-EHA enables additional depth curing. These monomers are opaque at wavelengths below 305/320 nm, and in particular the strong UVC line at 254 nm is limited to initiate polymerization in surface regions. The reduction in transmittance with increasing film thickness for PTHF_2900_-DM and 2-EHA is displayed in Figure 6a. Small differences in the absorption gain influence with increasing depth and have a considerable impact on curing. Phenol-based additives such as BHT show a tendency to yellowing, resulting in increased absorbance of PTHF_2900_-DM in 290–400 nm compared with 2-EHA [34].

PBS containing PTHF_2900_-DM and varying concentration of PI exhibit a strong reduction in transmittance below 420 nm, with a transmission maximum at 345 nm and a minimum at 375 nm, for PI concentration up to 0.55 wt % (Figure 6b). This reflects the characteristic absorbance pattern of the PI, as is concluded from spectra of neat TPO-L (10^−5^ M in ethanol). In case of PI concentration above 0.75 wt %, light between 420 and 200 nm is fully absorbed at one centimeter path length. The low absorptivity of the monomers, along with the unambiguous assignment of peaks in the transmittance spectra of formulations containing TPO-L, provide a promising basis for photobleaching experiments and give a reason to expect high curing depths.

### 3.2. Monitoring of Photobleaching

**Figure 7:**
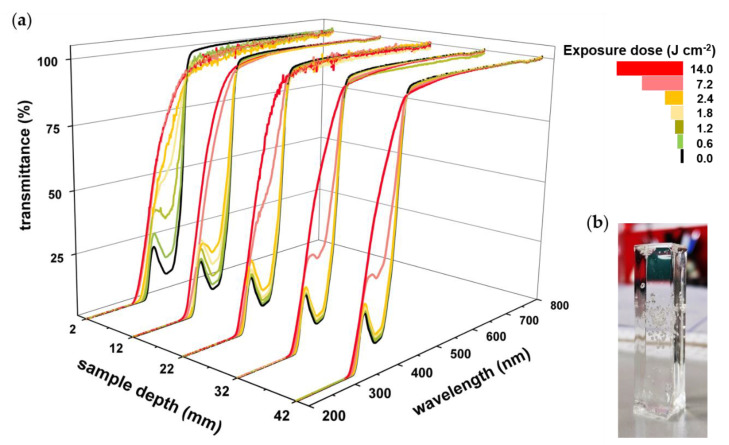
(**a**) Sample depth-dependent photobleaching of TPO-L in a bulk formulation containing PTHF_2900_-DM and 0.15 wt % PI. With increasing exposure dose and intensity, the transmittance between 420 and 320 nm increases from top (2 mm) to bottom (42 mm), reflecting progress in penetration depth and formation of free radicals through the entire sample depth. After the last irradiation cycle (total exposure 14.0 J cm^−2^) a uniform transmittance pattern of the 42 mm high specimen is achieved. (**b**) Cured specimen obtained after photopolymerization with an exposure dose of 14.0 J cm^−2^.

**Figure 8:**
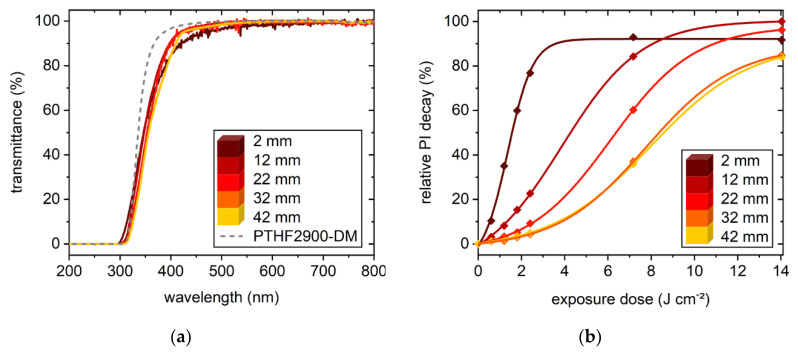
(**a**) Comparison of transmittance spectra at 2–42 mm after exposure with 14.0 J cm^−2^ indicates uniform bleaching; (**b**) normalized sample depth and exposure-dose-dependent photo initiator decomposition. With increasing dose, TPO-L decomposes gradually from top to bottom, ensuring progress in penetration depth and possibly high through-cure. At 42 mm depth, the maximum exposure dose leads to 84% decomposition (normalized to the maximum decomposition observed at a depth of 12 mm).

With an assembly enabling height-resolved collection of spectra, the correlation between photoinitiator decay and increased penetration depth was investigated by UV-Vis spectroscopy. Photobleaching was monitored for a PBS of PTHF_2900_-DM containing 0.15 wt % TPO-L. Due to characteristic peaks, low PI concentration proves to be ideal when monitoring photobleaching, although possibly not sufficient for homogenous curing. The maximum depth (44 mm) results from the height of the employed standard cuvette. Prior to exposure, transmittance spectra identically reflect the absorbance characteristics of TPO-L with a minimum in transmittance at 375 nm (Figure 7a). After the first irradiation cycle (0.6 J cm^−2^), an overall increase in transmittance between 420 and 320 nm occurs up to a depth of 12 mm. By enhancing the power level (intensity), the lower layers are reached as well (cycles 5 and 6, total exposure dose: 7.2 and 14.0 J cm^−2^). Finally, the spectra from top to bottom exhibit highly similar patterns converging to the spectrum of pure PTHF_2900_-DM (Figure 8a). Applying these settings (exposure system and PBS), it is possible to achieve photobleaching up to 42 mm depth, with a power level of 60% being sufficient to induce photoinitiator decay similar to that in near-surface regions. The clear specimen obtained from this experiment had reached gelation but was not tested in regard to homogenous curing because of the low photoinitiator concentration (Figure 7b).

Based on changes in transmittance at 375 nm, the relative course of bleaching/photoinitiator decay is traced in detail (Figure 8b). At maximum exposure, decay rates at 12 and 22 mm appear to exceed those close to the surface, so that PI degradation seems less effective at 2 mm depth (92%, normalized to the maximum decomposition observed at 12 mm). As discussed earlier, this finding is attributed to a slight yellowing caused by the oxidation of phenol-based additives during exposure.

### 3.3. Polymerization Behavior of Photobleaching Systems

Though photobleaching results are highly promising regarding PI decay and penetration depth, the experiments are not sufficient to provide information about the curing process itself. To gain an overview on polymerization behavior and cure depth, photobleaching systems A–F were investigated (Table 3). The used monomers differ greatly: PTHF_2900_-DM is of rather high viscosity (ca. 3500 Pa s) and implies a cross-linked polymer structure due to its difunctional nature, whereas 2-EHA serves as reactive diluent and generates linear polymers.

**Figure 9:**
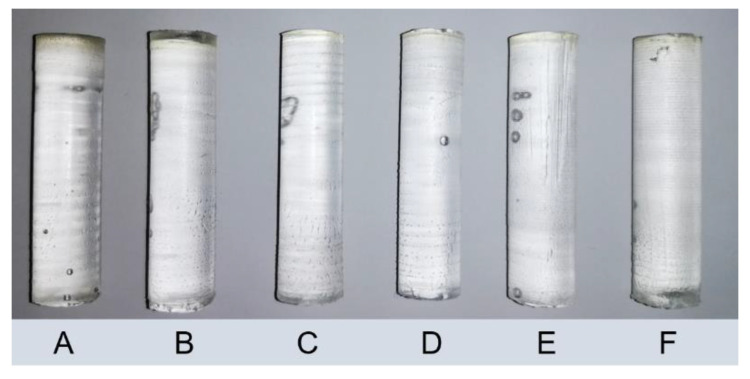
Depth cure of PBS A–F, and resulting specimens (height 52 mm, diameter 10 mm).

Analysis of the curing behavior of these PBS under uniform conditions should provide insight on the influence of monomer ratio and photoinitiator amount. To allow sufficient photobleaching, the TPO-L concentration does not exceed 1.0 wt %. The intensity profile is adapted to a total exposure dose of 20.8 J cm^−2^ (see also Section 2.5). After curing, 52 mm high, clear and uniform test specimens were released from the molds, sectioned and further characterized (Figure 9). Regardless of the PBS, a slight yellowing close to the surface was observed. It is assumed that the formation of quinones from BHT, triggered by UV irradiation and increased temperature, is responsible for the yellowing [33].

**Figure 10:**
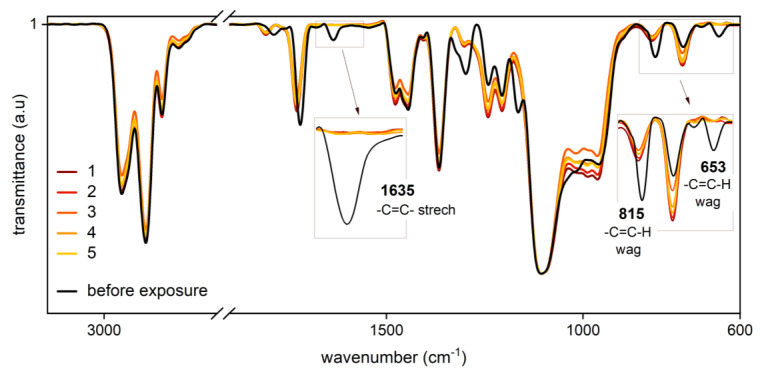
FT-IR spectroscopy of sections 1–5 (PBS A). The peaks related to carbon-carbon double bond units of the unpolymerized formulation at 1635, 815, and 653 cm^−1^ vanish in each section after light induced polymerization.

A first impression on through-cure was obtained through FT-IR spectroscopy of each section of the specimens (1–5, corresponding to the depth in cm). PBS A exhibits consistent double bond conversion as indicated by disappearance of the corresponding peaks at 1635 (C=C stretch) and 653 cm^−1^ (C–H out of plane bending vibration) (Figure 10). However, due to the high molecular mass of PTHF_2900_-DM, the peaks assignable to the double bond are poorly pronounced. A band appearing at 825 cm^−1^ after polymerization, which is primarily attributable to carbon-carbon skeletal vibration, superimposes the distinctive peak at 815 cm^−1^ (C–H out of plane bending vibration). Due to these difficulties and in view of the fact that all tested samples gave highly similar results, an exact quantification was omitted. Nevertheless, these results point to high double bond conversions.

**Figure 11:**
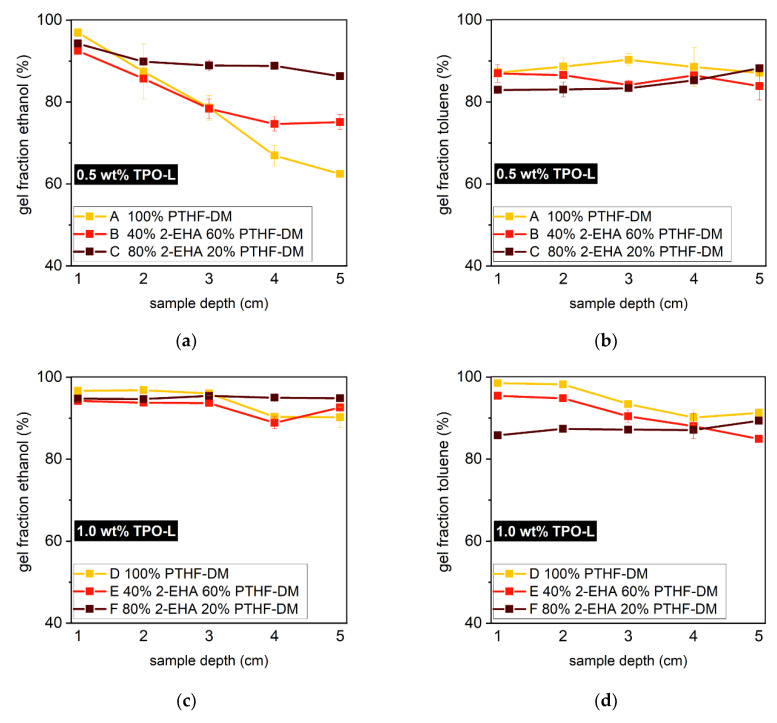
(**a**) Characteristics of the monomer composition play a key role in curing. For polymerization with 0.5 wt % TPO-L, a high quantity of 2-EHA appears beneficial. Gel contents below 90% are observed in toluene, though with high consistency (**b**). Regardless of the monomer composition and solvent, homogenous through-cure and uniform gel content are achieved using 1.0 wt % of photoinitiator (**c**,**d**).

A deeper understanding of depth-dependent curing is obtained by determination of the gel content, taking into account the whole sample volume. Considering polar and non-polar components, separate swelling tests in ethanol and toluene were carried out (Figure S1a–d). The resulting gel contents draw a more differentiated picture than initially suggested from FT-IR measurements. Both monomer ratio and TPO-L concentration play a vital role in homogenous through-cure: By comparing gel contents of PBS A–C (0.5 wt % TPO-L) in ethanol, a beneficial role of 2-EHA is suggested (Figure 11a). In PBS A the gel content decreases from 97% to 62%, whereas PBS C polymerizes much more homogeneously (top (1) 94% to bottom (5) 86%). These findings correlate with the UV-Vis data according to which 2-EHA features lower molar absorptivity than PTHF_2900_-DM. In contrast, gel contents in toluene are below 90%, though with higher consistency (Figure 11b). For photobleaching systems containing 1 wt % photoinitiator, the monomer ratio plays a subordinate role. Specimens D, E, and F exhibit gel contents above 90% along with excellent homogeneity (Figure 11c,d). Optimum conditions are met especially in PBS F, displaying uniform gel content from top to bottom, in both toluene and ethanol.

## 4. Conclusions

This study examines the applicability of photofrontal polymerization for the fast and easy production of (meth)acrylic polymers in bulk. Six photobleaching systems of varying monomer ratio (PTHF_2900_-DM and 2-EHA) and TPO-L concentration were cured under uniform conditions and characterized with regard to homogenous polymerization. UV-Vis spectroscopy serves to determine depth-dependent transmittance of monomers, featuring low optical density especially in the UVA and Vis region. By employing self-made inserts for the spectrophotometer, bleaching from top to bottom was tracked precisely and indicated the decisive role of the applied intensity for in-depth radical formation. Declining gel contents were found for photobleaching systems containing 0.5 wt % initiator, and the effect is alleviated with increasing 2-EHA ratio, possibly due to its lower molar absorptivity (up to 305 nm, compared to 320 nm for PTHF_2900_-DM). For photobleaching systems containing 1 wt % TPO-L, excellent curing down to 52 mm in depth was found, regardless of the monomer ratio. This study demonstrates how to accomplish fast and easy photofrontal polymerization up to 52 mm in depth by appropriately selecting parameters such as monomer composition, concentration of the photoinitiator, as well as intensity and type of the light source. After optimizing all influencing factors, curing with an even higher depth and curing of thick films with inferior optical properties seem likely.

## Figures and Tables

**Table 1:** Exposure profile applied in photobleaching experiments.

Cycle	Power Level (%)	Intensity (watt cm^−2^)	Runs	Exposure Dose (J cm^−2^)	Total Exposure Dose (J cm^−2^)
1	30	0.13	1	0.60	0.6
2	30	0.13	1	0.60	1.2
3	30	0.13	1	0.60	1.8
4	30	0.13	1	0.60	2.4
5	50	0.34	3	4.8	7.2
6	60	0.48	3	6.8	14.0

**Table 2:** Exposure profile applied for specimen production.

Cycle	Power Level (%)	Intensity (watt cm^−2^)	Runs	Exposure Dose (J cm^−2^)	Total Exposure Dose (J cm^−2^)
1	40	0.24	3	3.4	3.4
2	60	0.48	3	6.9	10.3
3	100	0.96	3	10.5	20.8

**Table 3:** Photobleaching systems for specimen production containing 0.5/1.0 wt % TPO-L and varying ratio of PTHF_2900_-DM and 2-ethylhexyl acrylate.

Photobleaching System	PTHF_2900_ Dimethacrylate (wt%)	2-EHA (wt%)	TPO-L (wt%)
A	100	0	0.5
B	100	0	1.0
C	60	40	0.5
D	60	40	1.0
E	20	80	0.5
F	20	80	1.0

## Supplementary Materials

The following are available online at https://www.mdpi.com/2073-4360/12/6/1291/s1, Figure S1a–d: Swelling ratios of specimens derived from PBS A–F in ethanol and toluene.
