# Supplementary material for: Ultra-High Through-Cure of (Meth)Acrylate Copolymers via Photofrontal Polymerization
Source: Polymers (Basel). 2020 Jun 4;12(6):1291. doi: 10.3390/polym12061291 (PMC7361706; doi:10.3390/polym12061291)
Supplement: Supplementary file 1 [file polymers-12-01291-s001.pdf]

polymers

MDPI

Supplementary

Ultra-High Through-Cure of (Meth)Acrylate Copolymers via Photofrontal Polymerization

Catharina Ebner [1], Julia Mitterer [1], Paul Eigruber [1], Sebastian Stieger [2], Gisbert Riess [1,*] and Wolfgang Kern [1]

[1] Department of Polymer Engineering and Science, Chair in Chemistry of Polymeric Materials, Montanuniversitaet Leoben, 8700 Leoben, Austria; catharina.ebner@unileoben.ac.at (C.E.); julia.mitterer@unileoben.ac.at (J.M.); paul.eigruber@unileoben.ac.at (P.E.); wolfgang.kern@unileoben.ac.at (W.K.)

[2] Department of Polymer Engineering and Science, Chair of Injection Moulding of Polymers, Montanuniversitaet Leoben, 8700 Leoben, Austria; sebastian.stieger@unileoben.ac.at

* Correspondence: gisbert.riess@unileoben.ac.at; Tel.: +43-3842-402-2301

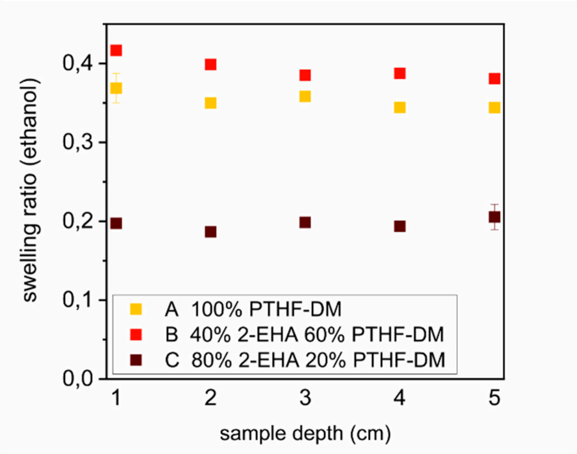

(**a**)

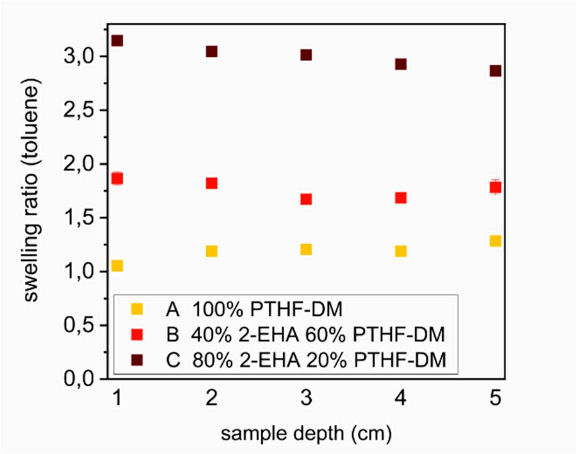

(**b**)

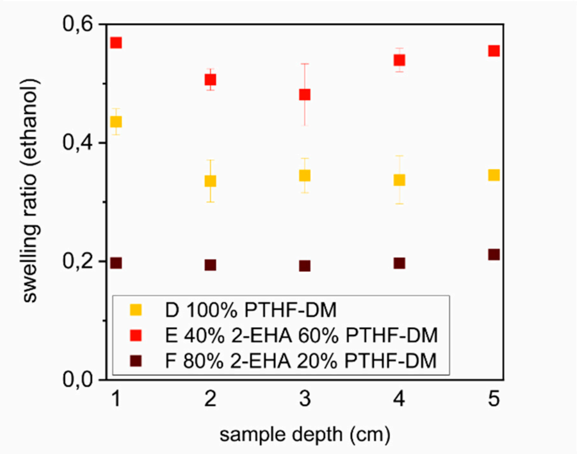

(**c**)

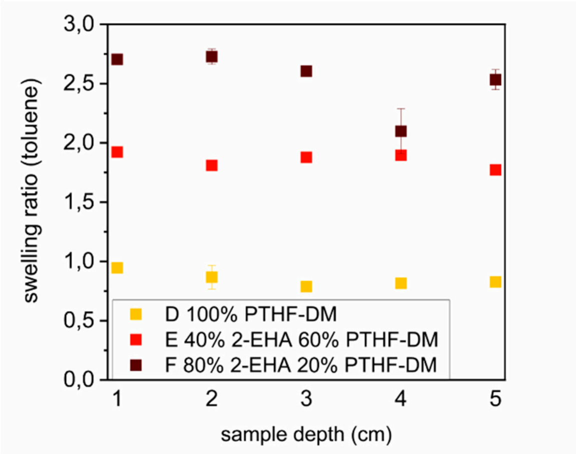

(**d**)

Figure S1. a–d: Swelling ratios of specimens derived from PBS A–F in ethanol and toluene .
